# An unusual presentation of a sarcoidosis that mimicked lymphatic metastatize non small cell lung carcinoma in positron emission tomography: a case report

**DOI:** 10.4076/1757-1626-2-6718

**Published:** 2009-09-15

**Authors:** Omer Dzemali, Nestoras Papadopoulos, Farhad Bakhtiary, Panagiotis Therapidis, Peter Kleine

**Affiliations:** Department of Thoracic and Cardiovascular Surgery of Johann Wolfgang Goethe University HospitalTheodor-Stern-Kai 7, 23A, 60590Frankfurt am Main/Germany

## Abstract

In the last decade, several sophisticated and accurate imaging methods such as positron emission tomography have been developed in order to evaluate malignant potential in enlarged mediastinal lymph nodes. This case illustrates an unusual presentation of sarcoidosis that mimicked lymphatic metastases of non small cell lung carcinoma. The reported high specificity and sensitivity of positron emission tomography-Computer Tomography regarding mediastinal staging could lead in same cases of false positives to a delaying of stage adapted therapy of non small cell lung carcinoma, showing that despite the recent advances of imaging techniques, such as positron emission tomography-computer tomography, several limitations of this imaging technique are still existing.

## Introduction

Accurate staging of mediastinal and hilar lymph nodes is a critical factor determining operability in patients with NSCLC. Positron emission tomography with has recently been reported to be more effective in detecting tumor involvement in mediastinal and hilar lymph nodes than computed tomography. We report about an interesting case of false-positive PET finding in mediastinal and hilar lymph node staging.

## Case presentation

**Figure 1.**
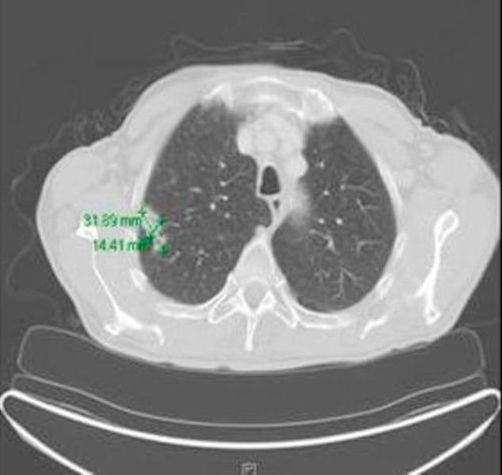
1.4 × 3.1 cm pulmonary mass in the lateral segment of the RUL associated with enlarged mediastinal lymph nodes.

**Figure 2.**
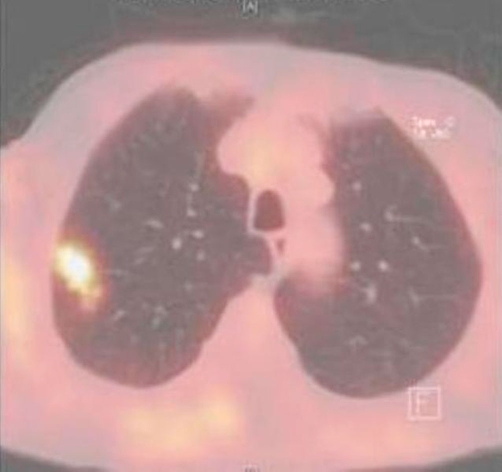
High fluorodeoxyglucose (FDG) uptake in the RUL lesion with maximum standartized uptake value (SUVmax) of 6.9.

**Figure 3.**
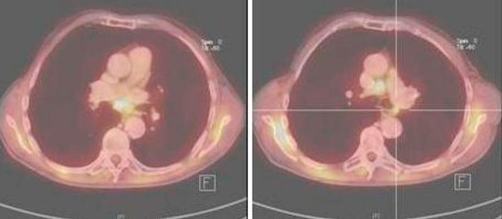
Further uptake was detected in precarinal (SUVmax: 6.4), infracarinal (SUVmax: 6.4) and paraaortal (SUVmax: 4.0) lymph nodes as well in a lymph node at the left main bronchus (SUVmax: 4,0), correspond to N3 disease.

An 80-year-old man (ethnic origin: German, nationality: German) with the medical history of Parkinson’s disease consulted his neurologist because of paresthesia in his lower extremities. The patient had 40 pack-years smoking history but had quit 26 years earlier. His medical history showed mitral valve insufficiency grade 2, hypertension and hypercholestererolemia. The social history, family history and review of systems were otherwise unremarkable. The physical examination revealed a generally light reduced state of health, normal vital signs, no fever, with bilaterally clear lung fields and no lymphadenopathy. His routine lab values, including complete blood count and chemistries showed elevated creatinine (1.87 mg/dl) and urea (93 mg/dl) as well as normochromic normocyteric anaemia. After readjustment of medical therapy, chest X-ray was done to exclude a paraneoplastic cause of the above-mentioned symptom. Chest X-ray revealed a solid contrast-enhancing mass in the right upper lobe (RUL). Based on these finding CT-scan was ordered and demonstrated a 1.4 × 3.1 cm pulmonary mass in the lateral segment of the RUL associated with 2.2 cm node in precarinal and 2.4 cm node paraaortal position (Figure 1). A flexible bronchoscopy and transbronchial biopsy of a parabronchial lymph node were performed. The endoscopic findings revealed no endobronchial lesion. Microbiological cultures of the gained bronchial secretion showed no evidence of the growth of germs, fungi or mycobacteria and the pathology report was negative for malignancy. CT-guided needle biopsy showed also no evidence of malignant tumour. PET-CT scan was performed to assess mediastinal nodal disease, and showed high fluorodeoxyglucose (FDG) uptake in the RUL lesion with maximum standardized uptake value (SUVmax) of 6.9 (Figure 2). Further uptake was detected in precarinal (SUVmax: 6.4), infracarinal (SUVmax: 6.4) and paraaortal (SUVmax: 4.0) lymph nodes as well in a lymph node at the left main bronchus (Figure 3) corresponding to N3 disease. The TNM classification after PET staging was NSCLC T2 N3 M0 (UICC IIIB). It was decided to proceed to thoracotomy in order to obtain histological confirmation. A right lateral thoracotomy was performed and wedge resection of the RUL. The pathologist’s intraoperative examination resulted in NSCLC (squamous cell carcinoma, T2) in the RUL. Additionally stage adapted lymph node dissection was performed. Surprisingly microscopic examination of the resected RUL tissue revealed except the squamous cell carcinoma signs of granulomatous inflammation. Numerous epitheloid cell granulomas were also found in all resected lymph nodes, which were free of malignancy. In summary, histological diagnosis was pulmonary sarcoidosis in addition to NSCLC. Resected margins and pleural surface were free of malignancy. The following TNM classification arose from the pathological examination: pT2, pN0, M0, G2, R0. Postoperative progress was satisfactory and the patient was discharged 7 days after surgery. Due to the reduced general state of health, no secondary lobectomy or adjuvant chemotherapy was indicated.

## Discussion

In recent years several sophisticated and accurate imaging methods have been developed in order to evaluate malignant potential in solitary pulmonary masses associated with enlarged mediastinal lymph nodes.

Multiple studies have demonstrated that PET-CT is more reliable as a single test for predicting malignancy in a radiologically undifferentiated nodule and in enlarged mediastinal lymph nodes than CT scan [1-3]. Positron emission tomography (PET) is a nuclear medicine imaging technique which takes advantage of the biological activity of tumour cells. The system detects pairs of gamma rays emitted indirectly by a positron-emitting radionuclide (tracer), which is introduced into the body on a biologically active molecule. Images of tracer concentration in 3-dimensional space within the body are then reconstructed by computer analysis. The most commonly used PET scanning isotope is 18F-FDG (F-fluoro-2-deoxy-D-Glucose); the concentrations of tracer imaged then give tissue metabolic activity, in terms of regional glucose uptake. The concept of emission and transmission tomography was introduced by David Kuhl and Roy Edwards in the late 1950s. Their work later led to the design and construction of several tomographic instruments. In the 1970s, Tatsuo Ido was the first to describe the synthesis of 18F-FDG.

The reported sensitivity and specificity of PET regarding mediastinal staging is reported to be 74-84% and 85-89% respectively with a negative predictive value of 93-95% [4,5]. Gould and coworkers reported about a sensitivity of even 100% and a specificity of 78% in particular in patients with in CT detected enlarged mediastinal lymph nodes [6]. On the other hand, in up to a quarter of patients wrongly positive findings may result. Therefore, histological confirmation of positive PET staging has to remain golden standard.

In our case a whole body PET-CT scan was performed to assess mediastinal nodal disease, and showed high FDG uptake in precarinal, infracarinal, paraaortal and contralateral left main bronchus lymph nodes corresponding to N3 disease (T2, N3, M0 / UICC IIIB). Transbronchial biopsy of a parabronchial lymph nodes and CT-guided needle biopsy of the pulmonary mass showed no evidence of malignant tumor. Afterwards we decided to proceed to thoracotomy in order to assess the high uptake lymph nodes and lesion in the RUL. The lymph nodes found to be positive on PET were discovered to be not malignant by the surgical biopsy. Numerous epitheloid cell granulomas were found in all resected lymph nodes in accordance with sarcoidosis. Pathologic staging resulted in significant downstaging relevant for further therapy, as our patient based on current ATS guidelines would have been a candidate for palliative chemotherapy according to PET staging.

Despite of sarcoidosis, false positive PET results have been reported to occur, most notably in association with further active lung diseases such as aspergillomas, active tuberculosis and abscesses [7]. Also false negatives are possible for example in case of carcinoids and in tumour less than 10mm in diameter [7,8].

This case illustrates an unusual presentation of sarcoidosis that mimicked metastatic NSCLC. The high reported specificity and sensitivity of PET-CT regarding mediastinal staging could lead in same cases to a delaying of stage adapted therapy of NSCLC, showing that despite the recent advances of imaging techniques, such as PET-CT, several limitations of this imaging technique are still existing. Histological confirmation of positive PET findings have to remain the golden standard of NSCLC staging.

## Abbreviations

18F-FDG: 18fluoro-fluoro-2-deoxy-D-Glucose
ATS guidelines: American Thoracic Society guidelines
FDG: fluorodeoxyglucose
NSCLC: non small cell lung carcinoma
PET: positron emission tomography
PET-CT: positron emission tomography-computer tomography
RUL: right upper lobe
SUV: standardized uptake value
TNM-classification: classification of malignant tumours
